# Metal-dependent cell death resistance contribute to lymph node metastasis of oral squamous cell carcinoma

**DOI:** 10.3389/fcell.2025.1541582

**Published:** 2025-02-27

**Authors:** Xuan-Hao Liu, Guang-Rui Wang, Nian-Nian Zhong, Zheng-Rui Zhu, Yao Xiao, Zheng Li, Lin-Lin Bu, Bing Liu

**Affiliations:** ^1^ State Key Laboratory of Oral & Maxillofacial Reconstruction and Regeneration, Key Laboratory of Oral Biomedicine Ministry of Education, Hubei Key Laboratory of Stomatology, School & Hospital of Stomatology, Wuhan University, Wuhan, China; ^2^ Department of Radiation and Medical Oncology, Zhongnan Hospital of Wuhan University, Wuhan, China; ^3^ Department of Oral & Maxillofacial - Head Neck Oncology, School and Hospital of Stomatology, Wuhan University, Wuhan, China

**Keywords:** oral squamous cell carcinoma, lymph node metastasis, metal-dependent cell death resistance, cathepsin-V, glutathione peroxidase 4, cyclin dependent kinase inhibitor 2A

## Abstract

**Objectives:**

Ferroptosis and cuproptosis can be summarized as metal-dependent cell death. This study aimed to explore the expression of metal-dependent cell death resistance (MCDR) characteristics in tumor cells of oral squamous cell carcinoma (OSCC) and to explore its relationship with lymph node metastasis (LNM).

**Methods:**

By integrating single-cell data of OSCC from public databases, an expression matrix comprising 127,149 cells was constructed. Gene set scores were calculated using the irGSEA package, and GO and KEGG analyses were performed to identify enriched pathways. The R package monocle3 was employed to calculate the cell trajectory and infer evolutionary patterns. The MuSiC2 package was employed to enable the evaluation of cell proportions. Cell-cell interaction information was analyzed using the CellChat package. The expression of cathepsin V (CTSV), glutathione peroxidase 4 (GPX4), and cyclin-dependent kinase inhibitor 2A (CDKN2A) was validated via immunohistochemistry and multiplex immunohistochemistry in oral mucosa (OM), non-metastatic primary tumors (nPT), and metastatic primary tumors (mPT). Additionally, R package oncoPredict was utilized to identify potential drug sensitivities.

**Results:**

The malignant cells in OSCC were divided into five subtypes, among which Epi_2 existed more in mPT and had higher MCDR characteristics. In addition, Epi_2 enriched multiple malignant-related pathways such as HEDGEHOG, NOTCH, and MYC. The spatial transcriptome and bulk RNA data verified that the proportion of Epi_2 in mPT was higher than that in nPT. Cell communication analysis showed that the effect of Epi_2 on endothelial cells was enhanced, which was mainly reflected in VEGFR and CXCL signaling pathways. Immunohistochemical results showed that the expression of Epi_2 characteristic markers CTSV and GPX4 in mPT was significantly higher than that in nPT. Multiplex immunohistochemical results showed that the co-expression cells of CTSV, GPX4 and CDKN2A in mPT were more than those in nPT. OSCC patients with high Epi_2 characteristics may have immunotherapy resistance and anti-EGFR treatment resistance. Doramapimod was identified as a sensitive drug.

**Conclusion:**

There is a type of malignant cells with characteristics of MDCR in OSCC, which is related to LNM and treatment resistance. It provides a predictive marker for early diagnosis of LNM.

## 1 Introduction

Oral squamous cell carcinoma (OSCC) originates from the oral cavity, and its 5-year survival rate is about 50% (Zhang D. et al., 2024). When the disease progresses to locally advanced stage, this proportion will decrease to 30% (Zanoni et al., 2019; Bray et al., 2024; Zeng et al., 2024). As the cancer advances, tumor cells have the potential to metastasize to adjacent lymph nodes (LNs) through the lymphatic vessels that encase the tumor (Cao et al., 2023; Li et al., 2024). Statistics show that approximately 44% of oral cancer patients develop lymph node metastasis (LNM), a condition that notably worsens their prognosis (Ho et al., 2017; Zanoni et al., 2019). At present, the preferred treatment for OSCC with suspected or confirmed LNM is neck LN dissection, but this will inevitably cause some postoperative complications, such as bleeding, nerve injury and so on (Yu et al., 2024). At the same time, the patient’s immune-active LNs will be removed indiscriminately during this process. With the ongoing advancements in immunotherapy and nano-drug delivery systems (Zhou et al., 2023), predicting LNM accurately and preserving LNs after metastasis in OSCC patients are important areas of research (Wang et al., 2023; Xu et al., 2024; Zhong et al., 2024). Our previous work established the “PUMP” principle, which outlines four key stages involved in the process of LNM: preparation, unleash, migration, and planting (Cao et al., 2023). However, due to the challenges in accurately capturing the dynamic processes of LNM, the specific mechanisms underlying LNM remain insufficiently understood.

In 2012, Dixon et al. first proposed a new form of non-apoptotic cell death, ferroptosis (Dixon et al., 2012). Ferroptosis is an iron-dependent form of death induced by iron accumulation and excessive lipid peroxidation (Ashoub et al., 2024; Feng et al., 2024; Jaeschke and Ramachandran, 2024). Ten years later, the concept of cuproptosis was also put forward. Cuproptosis is a copper-dependent death in which cells die due to the direct binding of copper to the lipoic acid acylation component of the tricarboxylic acid cycle and the aggregation of enzymes, leading to protein toxic stress and further inducing cell death (Ma et al., 2024). Currently, in addition to ferroptosis and cuproptosis, other metal ion-induced tumor cell death pathways remain poorly understood. Some scholars define ferroptosis and cuproptosis as forms of metal-dependent cell death (Kciuk et al., 2024). As research into metal-dependent cell death advances, tumor treatment strategies targeting metal-dependent cell death have gained increasing attention. Approaches such as chelating agents, metal ion carriers, and drug delivery systems have demonstrated significant potential and advantages in cancer therapy (Chen et al., 2022). Additionally, metal-dependent cell death signaling plays a crucial role in tumor development. For example, BACH1-induced ferroptosis was found to promote lymphatic metastasis of esophageal squamous cell carcinoma through the BACH1-SCD1-OA axis (Xie et al., 2023); expression of cuproptosis gene set has been found to affect the prognosis and immune status of patients with head and neck squamous cell carcinoma (Zheng et al., 2022). In summary, both cuproptosis and ferroptosis play crucial roles in tumorigenesis, progression, and treatment. However, the involvement of metal-dependent cell death in LNM of OSCC remains to be fully elucidated.

In this study, we characterized metal-dependent cell death resistance (MCDR) signatures based on ferroptosis- and cuproptosis-related inhibitory genes. By integrating single-cell datasets of OSCC, we identified a malignant epithelial cell subtype exhibiting MCDR characteristics. Spatial transcriptomics and The Cancer Genome Atlas (TCGA) data demonstrated that the proportion of this subtype among malignant epithelial cells was associated with LNM. Immunohistochemistry further validated the expression of the subtype’s characteristic genes in metastatic and non-metastatic primary tumors (PT). Additionally, analysis of immunotherapy and targeted therapy data from the Gene Expression Omnibus (GEO) database revealed a potential link between this subtype and treatment resistance. Drug sensitivity analysis identified potential therapeutic agents targeting this subtype.

## 2 Materials and methods

### 2.1 Data acquisition

Single-cell RNA sequencing (scRNA-seq) datasets and therapy-associated datasets of OSCC for this study were obtained from the GEO repository (https://www.ncbi.nlm.nih.gov/geo/). Specifically, for single-cell RNA (scRNA) data, we selected metastatic PT (mPT), non-metastatic PT (nPT), metastatic LN (mLN) and non-metastatic LN (nLN) data from GSE188737 (Quah et al., 2023), GSE195655 (Horny et al., 2023), GSE181919 (Choi et al., 2023) and GSE164690 (Janjic et al., 2022). A total of 127,149 cells were integrated. In addition, the spatial transcriptome dataset used in this study was from GSE208253 (Arora et al., 2023). At the same time, we selected immunotherapy-related datasets GSE179730 (Liu et al., 2021) and GSE195832 (Obradovic et al., 2022), and targeted therapy datasets GSE65021 (Bossi et al., 2016) and GSE84713 (Klinghammer et al., 2017) from the GEO database. RNA sequencing (RNA-seq) data from 229 OSCC patients were also obtained from TCGA database (https://portal.gdc.cancer.gov/) for further analysis. An immune checkpoint geneset with negative regulatory function was summarized from previous studies for subsequent expression verification.

### 2.2 Specimen source and tissue microarrays

PT, adjacent oral mucosa (OM), and LN tissues were collected from OSCC patients at the Department of Oral & Maxillofacial - Head Neck Oncology, School and Hospital of Stomatology, Wuhan University. The specimens were fixed in 4% paraformaldehyde and embedded in paraffin. Ethical approval for the study was obtained from the Ethics Committee of the Hospital of Stomatology, Wuhan University (2018LUNSHENZI#A24), in compliance with both international and national guidelines for ethical biomedical research, including those from the National Institutes of Health regarding human tissue usage. Informed consent was obtained from all participants. The tissue microarrays incorporated mPT, nPT, mLN, nLN, and OM samples.

### 2.3 Pre-processing and analysis of scRNA data

To ensure data quality, cells with mitochondrial gene expression exceeding 10% were excluded. Highly variable genes across cells were identified using the FindVariableFeatures function from the Seurat package (Version 4.3.0.1), with a threshold of 2,000 genes. We use the Harmony (Version 1.2.0) package to remove batch effects on different single-cell datasets. Dimensionality reduction was conducted via principal component analysis (PCA) and uniform manifold approximation and projection (UMAP). Cell clustering was achieved using the FindClusters function with an optimized resolution. Differentially expressed genes (DEGs) for each cell subgroup were determined using the FindAllMarkers function, and cell types were annotated based on the expression profiles of marker genes.

### 2.4 Functional enrichment analysis

Functional enrichment analysis of the identified DEGs was conducted using the Gene Ontology (GO) and Kyoto Encyclopedia of Genes and Genomes (KEGG) databases (http://www.genome.jp/kegg). The results were visualized with the “clusterProfiler” and “ggplot2” packages. Statistical significance was defined as a *P. adjust* value below 0.05.

### 2.5 InferCNV analysis

The chromosome copy number variation (CNV) profiles for each epithelial cell subtype were computed using the R package infercnv (version 1.14.2) (https://github.com/broadinstitute/inferCNV). The CNV score was determined as the sum of squares of CNV regions. T cells and B cells were designated as the normal reference cells for this analysis. A cutoff value of one was selected, and denoising was applied as part of the data processing pipeline.

### 2.6 Transcription factors analysis

Transcription factors analysis was performed using the module pyscenic (Version 0.12.1) with default parameters. Then, the results are extracted and read by R. Based on the ggplot2 package, the differential enrichment of transcription factors in five malignant cell subtypes was visualized. Transcription factors searches were constrained to regions within 10 kb of the transcription start site as the central point.

### 2.7 Metal-dependent cell death resistance signature

According to the results of Tsvetkov et al., we selected MTF1, GLS and CDKN2A as cuproptosis signal inhibotory genes (Tsvetkov et al., 2022). In addition, based on the GOBP_NEGATIVE_REGULATION_OF_FERROPTOSIS pathway in Molecular Signatures Database (MSigDB) (https://www.gsea-msigdb.org/gsea/msigdb), we selected eight genes (NQO1, SLC7A11, FTH1, GPX4, HMOX1, NFE2L2, AIFM2, SQSTM1) that negatively regulate ferroptosis signals. In this study, we defined these 11 genes as MCDR signatures and used them for subsequent scoring.

### 2.8 Gene set scoring

The R packages irGSEA (Version 2.1.5) was employed for gene set scoring. IrGSEA evaluates the performance of multiple gene set scoring methods applied to scRNA-seq data, including AUCell, UCell, singscore, ssGSEA, JASMINE, and Viper (Fan et al., 2024). In addition, the algorithm AddModuleScore was also used to calculate the expression of MCDR signatures in spatial transcriptome samples, and to calculate the expression score changes of four copper-iron death-related pathways during cell evolution. Gene sets related to HALLMARK, HYPOXIA, GLUTATHIONE_METABOLISM, POSITIVE_REGULATION_OF_REACTIVE_OXYGEN_SPECIES_BIOSYNTHETIC_PROCESS, and GLYCOLYSIS_GLUCONEOGENESIS were sourced from the MSigDB (Subramanian et al., 2005; Liberzon et al., 2015; Castanza et al., 2023).

### 2.9 Developmental trajectory inference

To explore potential lineage differentiation among malignant cell populations, trajectory analysis was conducted using the monocle3 algorithm (Version 1.3.1). A CellDataSet object was generated via the newCellDataSet function. Cell trajectories were determined with the orderCells function and visualized using the plot_cells function.

### 2.10 Cell-cell ligand-receptor communication analysis

The CellChat R package (Version 2.1.2) was employed to identify and visualize intercellular communication networks between malignant cells and mesenchymal cells using scRNA-seq data. The R package contains about 3,300 validated molecular interactions, including about 40% of secretory autocrine/paracrine signal interactions, about 17% of extracellular matrix receptor interactions, about 13% of cell-cell contact interactions, and about 30% of non-protein signals. A minimum of 10 cells per group was required for cell-cell communication analysis.

### 2.11 Spatial transcriptome analysis

Preliminary processing of spatial transcriptome data based on seurat package. The AddModuleScore algorithm is used to calculate the score distribution of the MCDR label in the sample, and the SpatialFeaturePlot is used to visualize the score results. In addition, based on the annotation results of the integrated OSCC single-cell data, the CARD package (Version 1.1) was used to annotate the spatial transcriptome samples (Ma and Zhou, 2022). CARD is a reference-based deconvolution method. Its key feature is that it can adapt to the spatial correlation of cell type composition across tissue locations, so as to achieve accurate and spatial information-rich cell type deconvolution and fine spatial map construction. CARD relies on effective optimization algorithms with constrained maximum likelihood estimation, and can be extended to spatial transcriptomics with tens of thousands of spatial locations and tens of thousands of genes. Among them, the proportion of Epi_2 calculated by all spots of each sample is added, divided by the total proportion of epithelial cells, which is the proportion of Epi_2 in the sample.

### 2.12 Single-cell deconvolution analysis

MuSiC2 package (Version 0.1.0) is an iterative algorithm that aims to improve the cell-type deconvolution of batch RNA-seq data. By removing cell-type-specific DEGs between samples with different clinical conditions from a single-cell reference, MuSiC2 has the potential to produce more accurate cell-type proportion estimates. In this study, based on the integrated OSCC malignant cell subgroup annotation results, the TCGA data and treatment-related data were deconvolutionally evaluated for the proportion of malignant cells, and the proportion of Epi_2 in malignant cells of each patient was calculated. All patients were divided into Epi_2 high and Epi_2 low groups according to the median of Epi_2 proportion, and the immune infiltration of the two groups was evaluated using the CIBERSORT package (Version 0.1.0).

### 2.13 Prediction of therapeutic efficacy

MuSiC2 was used to predict the proportion of Epi_2 in patients in several treatment-related datasets. According to the treatment response of each patient to the drug, they were divided into response group and non-response group. The pROC package (Version 1.18.0) was used to draw the ROC curve to characterize the predictive ability of Epi_2 proportion for the effect of different treatment methods on patients. Among them, the larger the area under curve (AUC), the stronger the predictive ability.

### 2.14 Drug sensitivity analysis

Drug sensitivity analysis was carried out using the “oncoPredict” package, which utilizes data from 198 compounds in the Genomics of Drug Sensitivity in Cancer database. Drugs were screened based on *P* < 0.05 and Log2FoldChange >0.

### 2.15 Immunohistochemical staining

After paraffin embedding, tissue samples were sectioned into 4-um slices and mounted on glass slides. The sections were baked, dewaxed, and hydrated before antigen retrieval using sodium citrate solution (#G1201-1L, Servicebio, Wuhan, China). The slides were incubated with primary antibodies for cathepsin-V (CTSV) (1:100 dilution, rabbit, #A7662, ABclonal, Wuhan, China), glutathione peroxidase 4 (GPX4) (1:100 dilution, rabbit, #A1933, ABclonal, Wuhan, China), and cyclin dependent kinase inhibitor 2A (CDKN2A) (1:100 dilution, mouse, #A20371, ABclonal, Wuhan, China) followed by incubation with appropriate secondary antibodies. Staining was performed using DAB (#2310310031G, Maixin, Fuzhou, China) and hematoxylin (#G1004, Servicebio, Wuhan, China). The histo-score (H-score) was calculated using the formula: H-score = [(1+) × 1 + (2+) × 2 + (3+) × 3]. The sections were then sealed and scanned using an Aperio ScanScope CS scanner (Sausalito, CA, United States).

### 2.16 Multiplex immunohistochemical staining

Paraffin-embedded tissue slides were blocked by 3% BSA (#GC305006, Servicebio, Wuhan, China) and incubated overnight at 4^o^C with primary antibodies against CTSV. Subsequently, HRP-labeled secondary antibody (#GB23204, Servicebio, Wuhan, China) was added and incubated at room temperature for 50 min. Add iF488-TSA (#G1233, Servicebio, Wuhan, China) and incubate at room temperature in dark for 10 min. Subsequently, the antibody was eluted and blocked with 3% BSA again. The above steps were repeated, GPX4 and CDKN2A were incubated in turn, and iF555-TSA (#G1231, Servicebio, Wuhan, China) and iF647-TSA (#G1232, Servicebio, Wuhan, China) were added in turn. Cell nuclei were stained using 4′,6-diamidino-2-phenylindole (DAPI) (#P0131, Beyotime, Shanghai, China). The stained tissues were visualized using confocal microscopy (Olympus, Tokyo, Japan).

### 2.17 Statistical analyses

All bioinformatic analyses were conducted using R software (Version 4.2.3) and python (Version 3.8). Statistical analyses were performed using GraphPad Prism 10 (GraphPad Software, San Diego, CA, United States). Data are presented as the mean ± standard deviation (SD). Differences between two groups were assessed using paired or unpaired Student’s t-tests, while comparisons involving more than two groups were analyzed using one-way ANOVA. Spearman correlation and Pearson Chi-squared tests were used to assess correlations between variables. Statistical significance was set at *P* < 0.05.

## 3 Results

### 3.1 Integration of single-cell datasets and screening of malignant cells

**FIGURE 1:**
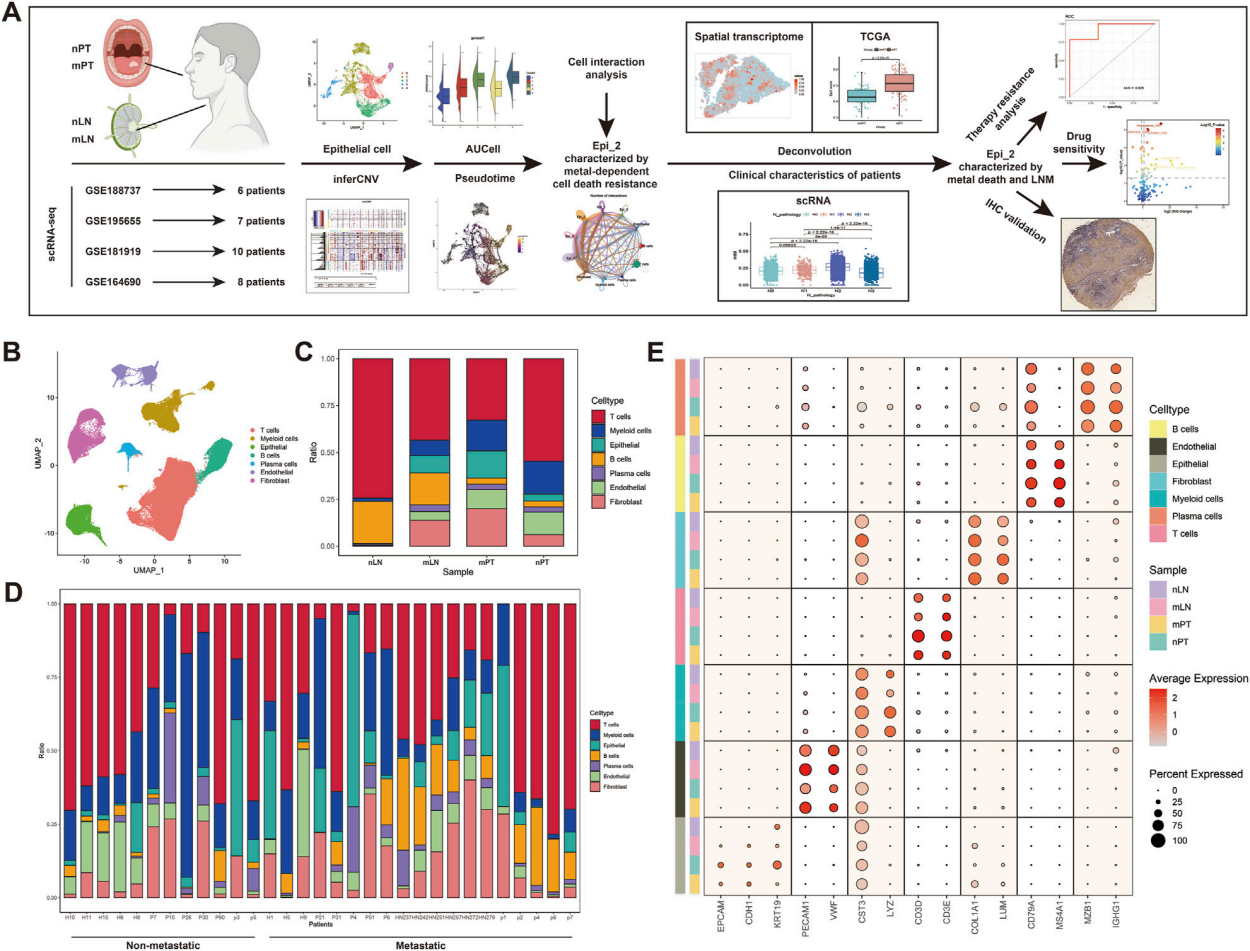
Integration and preliminary analysis of oral squamous cell carcinoma (OSCC) single-cell data. **(A)** The work flow chart of this study. **(B)** The UMAP of the integrated single-cell data. **(C)** The proportion of various cell types in non-metastatic lymph node (nLN), metastatic lymph node (mLN), metastatic primary tumor (mPT) and non-metastatic primary tumor (nPT). **(D)** The proportion of various cell types in all patients. **(E)** Expression dotplot of markers of various cell types.

We summarized four groups of single-cell datasets and briefly depicted the workflow of this study (Figure 1A). Subsequently, based on the common markers of various cells, we divided all cells into seven subclusters, including epithelial cells (EPCAM, CDH1, KRT19), endothelial cells (PECAM1, VWF), myeloid cells (CST3, LYZ), T cells (CD3D, CD3E), fibroblasts (COL4A1, LUM), B cells (CD79A, MS4A1) and plasma cells (MZB1, IGHG1) (Figure 1E). After the annotation was completed, we plotted the UMAP of all cells (Figure 1B), and showed the distribution and proportion of these seven types of cells in different samples (nLN, mLN, mPT, nPT) (Figure 1C). The results showed that T cells and B cells were dominant in nLN, and the proportion of epithelium and fibroblasts in mPT was significantly higher than that in nPT, while the proportion of T cells was relatively low, suggesting the potential relative “immune-desert” characteristics in mPT. Then, we showed the proportion of all types of cells in different patients (Figure 1D). In order to screen malignant cells, we took out epithelial cells from all cell types for inferCNV analysis (Supplementary Figure S1) and finally identified 10,260 cells as malignant epithelial cells for subsequent analysis.

### 3.2 Malignant cells in oral squamous cell carcinoma have obvious heterogeneity

**FIGURE 2:**
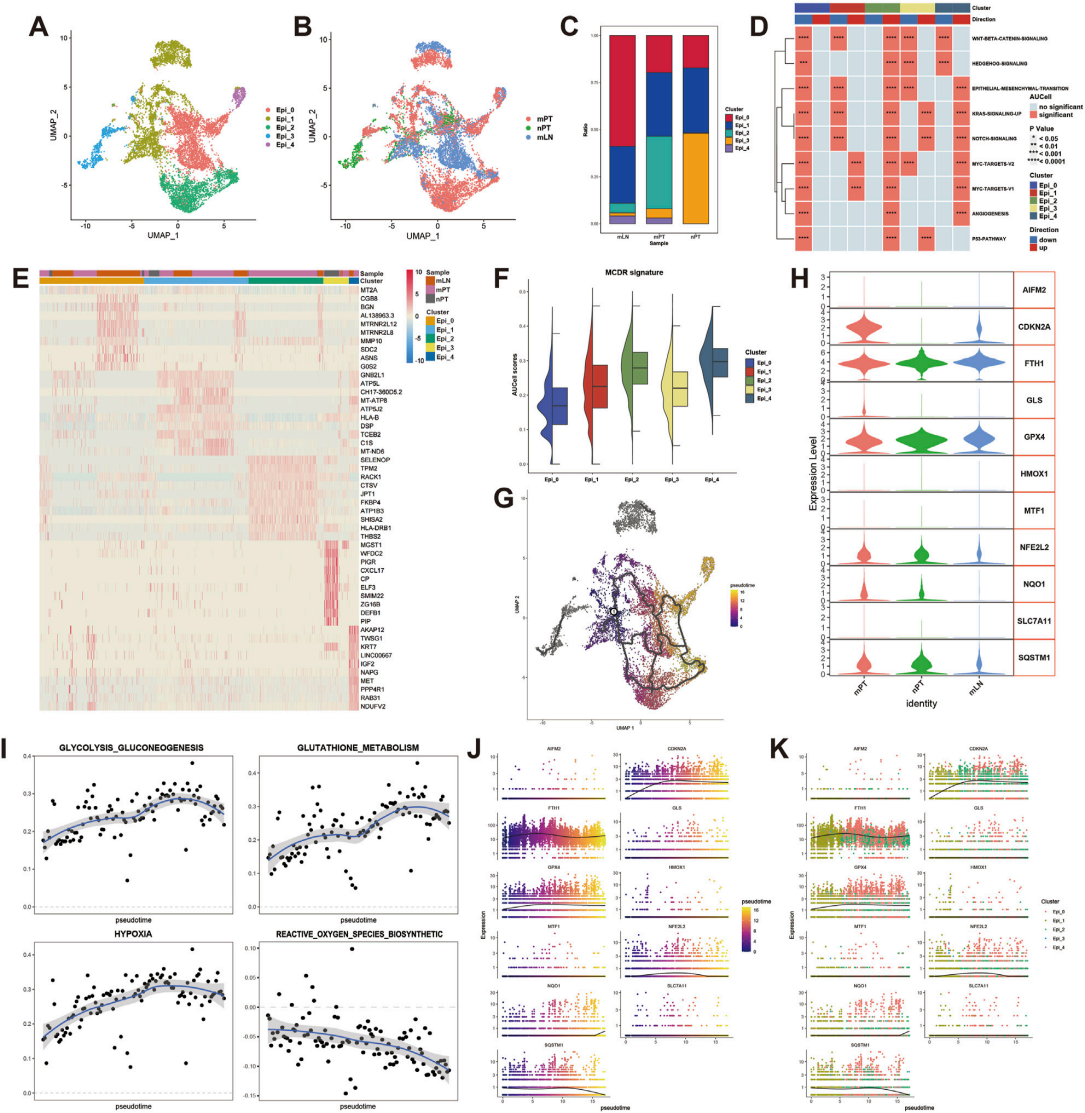
The presence of malignant cell subtype with the characteristics of metal-dependent cell death resistance (MCDR) in metastatic primary tumor. **(A)** The UMAP showing the clustering of malignant cells in oral squamous cell carcinoma (OSCC). **(B)** The UMAP showing the malignant cell distribution of metastatic primary tumor (mPT), non-metastatic primary tumor (nPT) and metastatic lymph node (mLN). **(C)** The proportion of five malignant cell subtypes in mLN, mPT and nPT. **(D)** HALLMARK pathway enrichment in malignant cells. **(E)** Expression heatmap of top10 differentially expressed genes of five malignant cells. **(F)** AUCell scores assessing MCDR characteristics in five malignant cell Subtypes. **(G)** The UMAP revealing the evolutionary trajectory in malignant cells. **(H)** The expression of MCDR characteristic genes in mPT, nPT and mLN. **(I)** The expression intensity changes of four signaling pathways related to MCDR during the evolution from Epi_1 to Epi_2. **(J)** Expression changes of MCDR characteristic genes with pseudo-time changes. **(K)** The expression changes of MCDR characteristic genes with the evolution of malignant cell subtypes.

We first performed UMAP dimensionality reduction on all malignant cells and divided them into five subtypes (Figures 2A,B). GO and KEGG enrichment results showed that these subtypes had certain functional heterogeneity (Supplementary Figure S2, S3). Among them, Epi_0 specifically enriched in Antigen processing and presentation, Epi_1 specifically enriched in Estrogen signaling pathway, Epi_2 specifically enriched in Apoptosis, Epi_3 specifically enriched in TNF signaling pathway, Epi_4 specifically enriched in Cellular senescence. At the same time, the expression heatmap of the top10 differential genes in each subtype and transcription factor analysis showed that each subtype had different gene expression and regulation patterns (Figure 2E; Supplementary Figure S4). Among them, Epi_2 accounts for the largest proportion of mPT (Figure 2C). The results of HALLMARK score showed that Epi_2 enriched a variety of classical pathways of malignant cells, including NOTCH pathway, HEDGEHOG pathway, MYC pathway, etc., and had a high potential for epithelial-mesenchymal transition (Figure 2D).

### 3.3 Epi_2 is associated with metal-dependent cell death resistance

In order to explore the characteristics of MCDR in malignant cells in OSCC, we screened 11 markers involved in the negative regulation of cuproptosis and ferroptosis, and defined them as MCDR signatures. Subsequently, we examined the expression of these 11 markers in three samples (Figure 2H). The signature scoring results of the five subgroups showed that Epi_2 and Epi_4 had higher MCDR characteristics (Figure 2F). In addition, Epi_2 simultaneously enriched multiple classical pathways of malignant cells, and the proportion of Epi_4 cells was relatively low, so we chose Epi_2 for further in-depth study. The results of pseudo-time analysis showed that there may be a process of transition from Epi_1 with low activity of MCDR to Epi_2 (mainly in mPT) with higher activity in OSCC (Figure 2G). In order to verify the change of MCDR activity, we evaluated the activity changes of four pathways in the transition from Epi_1 to Epi_2 (Figure 2I). The results showed that with the transition of Epi_1 to Epi_2, the activities of glycolysis, glutathione metabolism and hypoxia were enhanced, while the biosynthesis process of reactive oxygen species was gradually weakened, confirming the change of MCDR activity during the transition process. Subsequently, the results of pseudo-time analysis showed that among the 11 MCDR markers, GPX4 and CDKN2A were significantly increased during the transition from Epi_1 to Epi_2 (Figures 2J,K).

### 3.4 Epi_2 and metal-dependent cell death resistance are related to lymph node metastasis

**FIGURE 3:**
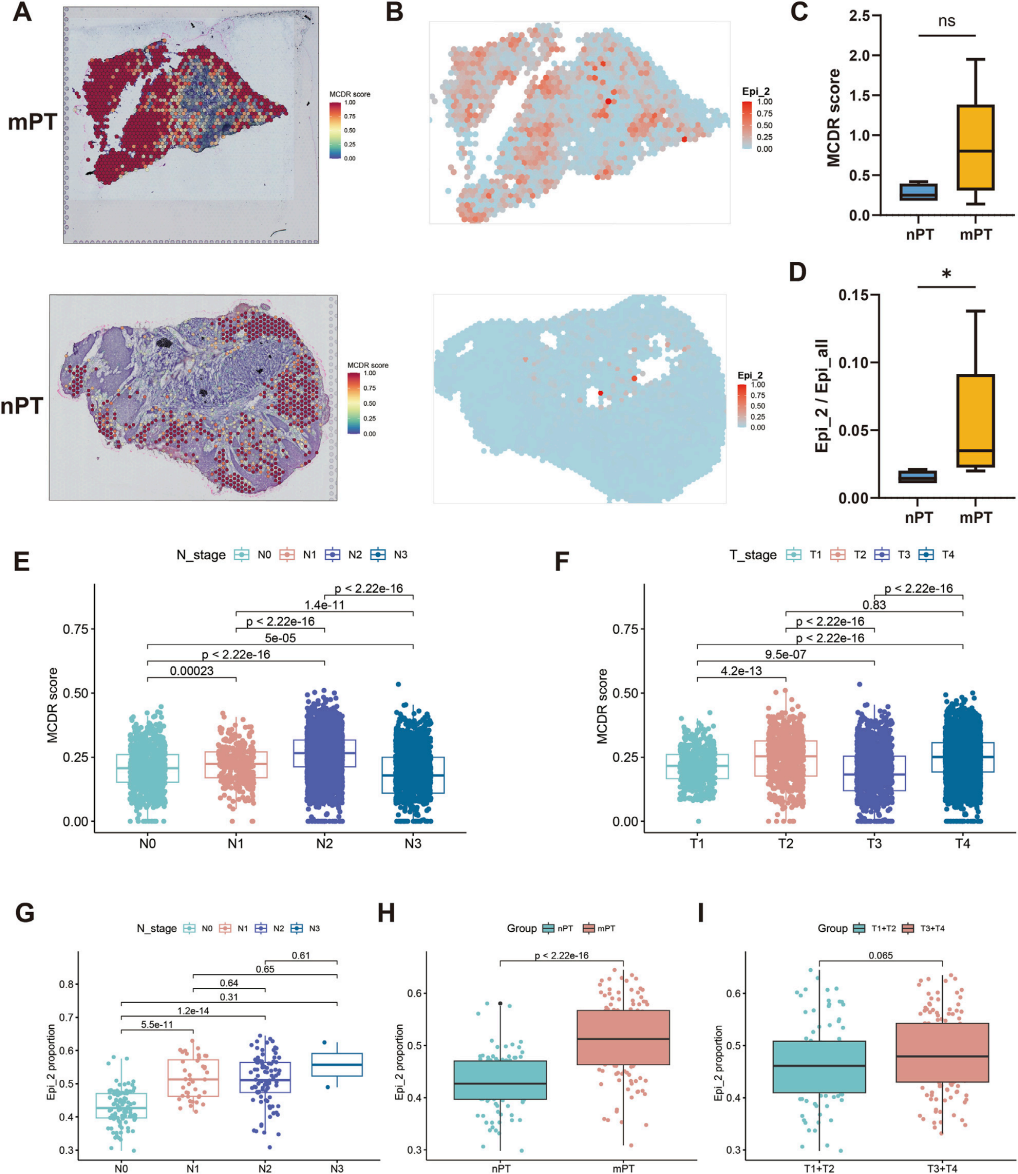
Epi_2 and metal-dependent cell death resistance (MCDR) characteristics are related to lymph node metastasis. **(A)** The distribution of MCDR scores in metastatic primary tumor (mPT) and non-metastatic primary tumor (nPT). **(B)** The distribution of Epi_2 in mPT and nPT. **(C)** Comparison of MCDR scores between nPT and mPT (*P* > 0.05). **(D)** Comparison of proportion of Epi_2 between nPT and mPT (*P* < 0.05). **(E)** Comparison of MCDR scores of malignant cells at different N stages in single-cell data. **(F)** Comparison of MCDR scores of malignant cells at different T stages in single-cell data. **(G)** Comparison of proportion of Epi_2 in OSCC with different N stages in TCGA data. **(H)** Comparison of the proportion of Epi_2 between nPT and mPT in TCGA data (*P* < 0.0001). **(I)** Comparison of the proportion of Epi_2 between T1T2 and T3T4 stage in TCGA data (*P* = 0.065). *P < 0.05, ns indicates not significant.

The MCDR scores of spatial transcriptome samples were evaluated. The results showed that the MCDR characteristics in mPT were richer than those in nPT (Figure 3A), but the difference between the two was not significant (*P* > 0.05) (Figure 3C). In addition, it was found that Epi_2 in mPT accounted for a higher proportion in the malignant cells compared with nPT (Figure 3B), and the difference was significant (*P* < 0.05) (Figure 3D). Subsequently, the MCDR scores of malignant cells in different N stages were further counted in single-cell data. The results showed that from N0 to N2 stage, the MCDR score gradually increased, while the N3 stage decreased significantly (Figure 3E). For different T stages, the MCDR score only showed a significant increase from T0 to T1 (*P* < 0.0001) (Figure 3F). The deconvolution evaluation results of OSCC samples in TCGA database showed that the proportion of Epi_2 in mPT was significantly higher than that in nPT (*P* < 0.0001) (Figure 3H), and there was no obvious difference in the proportion of Epi_2 in different N stages of mPT (Figure 3G). The median proportion of Epi_2 in OSCC of T3T4 stage was higher than that of T1T2 stage, but the difference was not statistically significant (*P* = 0.065) (Figure 3I).

### 3.5 Epi_2 has extensive communication with mesenchymal cells

**FIGURE 4:**
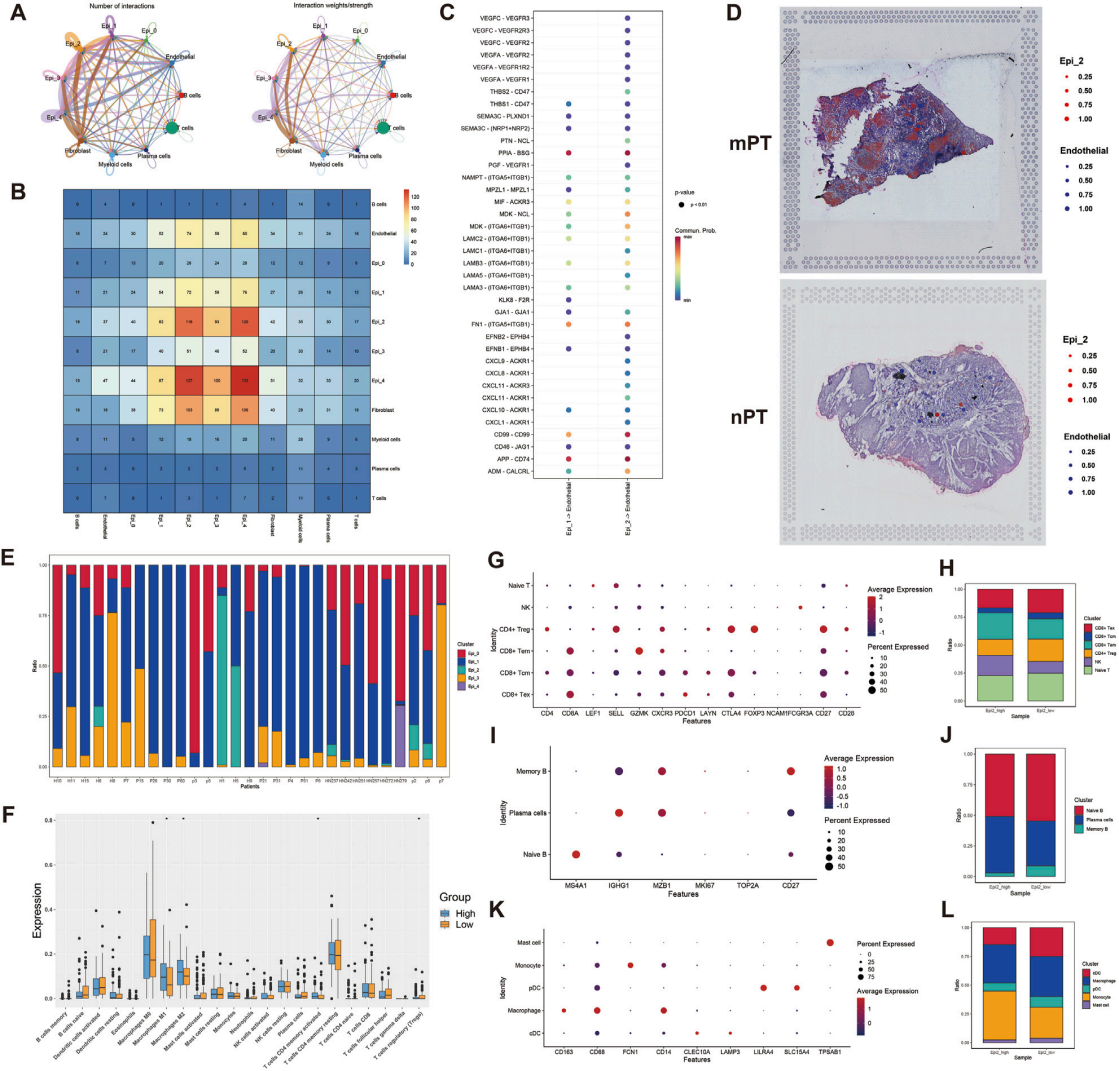
The effect of Epi_2 on tumor microenvironment. **(A, B)** The intensity and number of interactions between malignant epithelial cell subtypes and mesenchymal cells. **(C)** The receptor-ligand interaction of Epi_1 and Epi_2 on endothelial cells. **(D)** The spatial relationship between Epi_2 and endothelial cells in mPT and nPT. **(E)** Proportion of malignant epithelial cells in all patients. **(F)** The immune infiltration of patients with high Epi_2 and low Epi_2 in TCGA data. **(G)** The expression dotplot of the markers of each subtype of T cells. **(H)** The proportion of T cell subtypes in Epi_2 high and Epi_2 low groups. **(I)** The expression dotplot of the markers of each subtype of B cells. **(J)** The proportion of B cell subtypes in Epi_2 high and Epi_2 low groups. **(K)** The expression dotplot of the markers of each subtype of myeloid cells. **(L)** The proportion of myeloid cell subtypes in Epi_2 high and Epi_2 low groups.

The results of cell communication analysis showed that Epi_2 had higher interaction intensity with other mesenchymal cells than Epi_1 (Figure 4A). Among all mesenchymal cells, Epi_2 has the strongest communication with fibroblasts, followed by endothelial cells (Figure 4B). In view of the more obvious difference in the ligand-receptor effect between Epi_2 and endothelial cells, we chose endothelial cells as the object of subsequent analysis. Further comparison of the effect of Epi_1 and Epi_2 on endothelial cells, it was found that Epi_2 showed a stronger effect on VEGF and CXCL signaling pathways (Figure 4C). At the same time, in mPT, Epi_2 also showed a closer connection with endothelial cells in the spatial dimension (Figure 4D).

### 3.6 Immune microenvironment landscape of oral squamous cell carcinoma with Epi_2 feature

Based on the single-cell integrated data, the proportion of malignant cell subtypes in all patients was displayed (Figure 4E). According to the proportion of Epi_2, eight patients who expressed Epi_2 features were included in the Epi_2 high group, and the other patients were in the Epi_2 low group. At the same time, the immune infiltration analysis of TCGA data showed that the infiltration of M1 (*P* < 0.05) and M2 (*P* < 0.05) macrophages in the tumors with high Epi_2 was significantly increased (Figure 4F). Subsequently, the immune cells in the single-cell data were subdivided. For T cells, they are divided into naive T cells, natural killer (NK) cells, CD4^+^ regulatory T cells (Treg), CD8^+^ effector memory T cells (Tem), CD8^+^ central memory T cells (Tcm) and CD8^+^ exhausted T cells (Tex) according to their expression markers in different functional states (Figure 4G), and found that the proportion of CD8^+^ Tex in the Epi_2 high group was relatively low (Figure 4H). For B cells, they were divided into memory B cells, plasma cells, and naive B cells (Figure 4I), and found that the proportion of plasma cells in the Epi_2 high group was relatively high (Figure 4J). For myeloid cells, they were divided into mast cells, monocytes, plasmacytoid dendritic cells (pDC), macrophages and conventional dendritic cells (cDC) (Figure 4K), and it was found that the proportion of cDC in the Epi_2 high group was lower than that in the Epi_2 low group (Figure 4L).

### 3.7 Expression of Epi_2 characteristic markers is related to lymph node metastasis

The expression of the top 8 DEGs of Epi_2 in the three groups of samples showed that the expression of CTSV in mPT was significantly higher than that of nPT and mLN (Supplementary Figure S5A), and the expression of CTSV was positively correlated with the MCDR score of malignant cells (*P* < 0.0001, r = 0.23) (Supplementary Figure S5B). Combined with the results of the previous pseudotime analysis, CTSV, GPX4 (ferroptosis inhibitory gene) and CDKN2A (cuproptosis inhibitory gene) were defined as characteristic markers of Epi_2.

**FIGURE 5:**
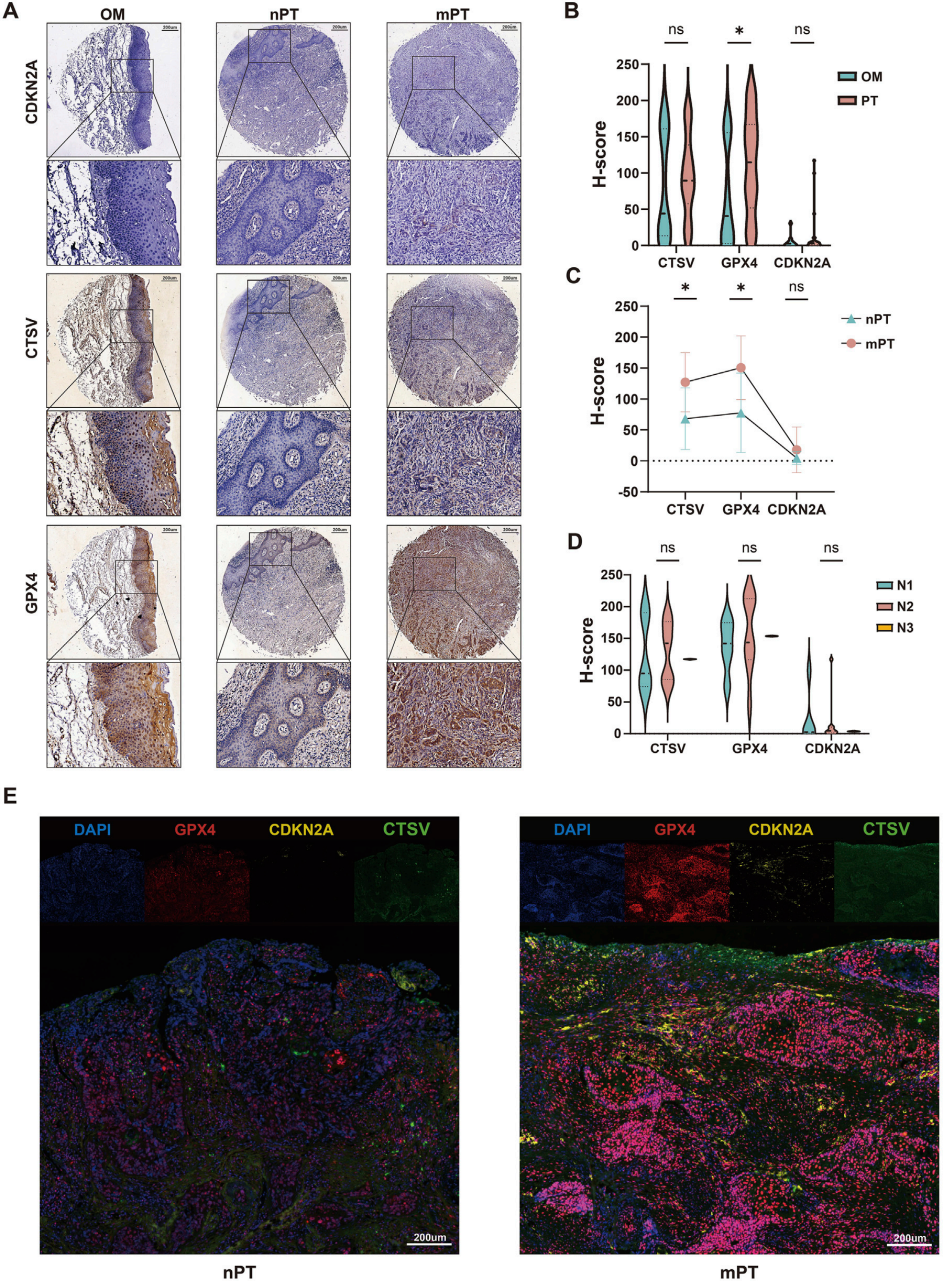
The expression of Epi_2 characteristic markers CTSV, GPX4 and CDKN2A in oral squamous cell carcinoma (OSCC). **(A)** CDKN2A, CTSV and GPX4 expression in oral mucosa (OM), metastatic primary tumor (mPT) and non-metastatic primary tumor (nmPT), respectively. Scale bar: 200 µm. **(B)** The expression differences of the three in OM and OSCC. **(C)** The expression differences of the three in nPT and mPT. **(D)** The expression differences of the three in N1, N2 and N3 stage of OSCC. **(E)** The fluorescence co-expression of the three in nPT and mPT. Scale bar: 200 µm *P < 0.05, ns indicates not significant.

Subsequently, the expression of CTSV, GPX4 and CDKN2A was detected by tissue microarray (Figure 5A). The results showed that the expression of GPX4 in OSCC was significantly higher than that in OM (*P* < 0.05), and there was no significant difference in the expression of CTSV (*P* > 0.05) and CDKN2A (*P* > 0.05) (Figure 5B). At the same time, the expression of CTSV (*P* < 0.05) and GPX4 (*P* < 0.05) in mPT was higher than that in nPT, and there was no significant difference in CDKN2A (*P* > 0.05) (Figure 5C). When the expression of the three in different N stages was counted, the results showed that there was no significant difference in the expression of the three in N1, N2 and N3 stage (*P* > 0.05) (Figure 5D). The results of multiplex immunohistochemical showed that GPX4 was mainly expressed in the nucleus, and CDKN2A and CTSV were mainly expressed in the cytoplasm (Figure 5E). At the same time, there are more CTSV, GPX4 and CDKN2A co-expressed cells in mPT than in nPT.

### 3.8 Epi_2 is associated with resistance to immunotherapy and EGFR targeted therapy

**FIGURE 6:**
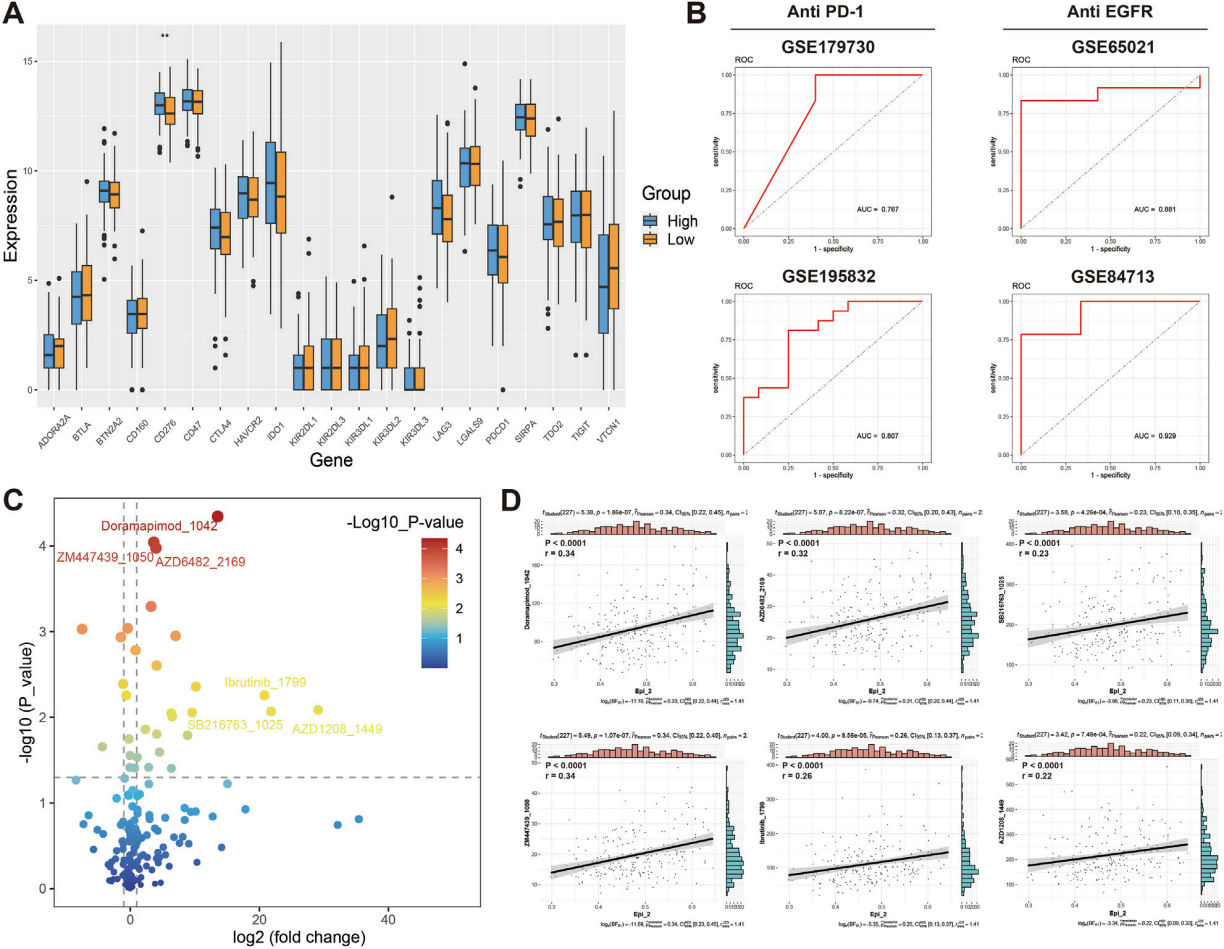
Analysis of treatment resistance and drug sensitivity in patients with high Epi_2 feature. **(A)** The expression difference of immune checkpoint genes in OSCC with high Epi_2 and low Epi_2 feature in TCGA data. **(B)** In the data of immunotherapy (AUC = 0.767, AUC = 0.807) and anti-EGFR targeted therapy (AUC = 0.881, AUC = 0.929), patients with high Epi_2 feature may show resistance to both treatments. **(C)** Volcano plot show all candidate sensitive drugs for patients with high Epi_2 feature. **(D)** The correlation between drug sensitivity and Epi_2 features of six candidate drugs. **P < 0.01.

The expression of 21 immune checkpoint genes in OSCC of Epi_2 high and Epi_2 low groups in TCGA data was detected (Figure 6A). The results showed that only CD276 was significantly highly expressed in OSCC of Epi_2 high group (*P* < 0.01), and there was no significant difference in the expression of other indicators (*P* > 0.05). In addition, the correlation between Epi_2 feature and treatment response was evaluated, and it was found that OSCC patients with high Epi_2 feature may have certain therapeutic resistance to immunotherapy (AUC = 0.767, AUC = 0.807) and anti-EGFR therapy (AUC = 0.881, AUC = 0.929) (Figure 6B).

### 3.9 Doramapimod is a candidate drug for the treatment of OSCC patients with high Epi_2 feature

The drug sensitivity analysis of two groups of OSCC in TCGA data was performed, and all sensitive drugs of OSCC patients with high Epi_2 feature were displayed (Figure 6C). The three drugs with the smallest *P* value and the three drugs with the largest Log2FoldChange were Doramapimod (*P* < 0.0001, r = 0.34), ZM447439 (*P* < 0.0001, r = 0.34), AZD6482 (*P* < 0.0001, r = 0.32), lbrutinib (*P* < 0.0001, r = 0.26), SB216763 (*P* < 0.0001, r = 0.23) and AZD1208 (*P* < 0.0001, r = 0.22), respectively, and their correlation with Epi_2 expression was analyzed (Figure 6D). Combined with the above parameters, Doramapimod was finally determined as a candidate therapeutic drug for OSCC patients with high Epi_2.

## 4 Discussion

LNM is a dynamic and multifaceted process. Our previous research outlined several mechanisms underlying LNM, namely, the “PUMP” principle (Cao et al., 2023). The process begins with the “Preparation” stage, characterized by the establishment of an immunosuppressive microenvironment and the promotion of lymphangiogenesis. This is followed by the “Unleash” phase, during which tumor cell adhesion decreases and the extracellular matrix is degraded. The next step is the “Migration” stage, where tumor cells actively migrate and lymphatic endothelial permeability increases. Finally, in the “Planting” stage, tumor cells adhere to and colonize the lymph nodes, accompanied by enhanced proliferative capacity. Due to the dynamic nature of LNM, no scRNA-seq data currently exists to comprehensively analyze its dynamic process. This study focuses on the characteristics of MCDR and identifies a subset of malignant cells associated with the “Preparation” stage. These cells may contribute to lymphangiogenesis and the establishment of an immunosuppressive microenvironment. Furthermore, the study explores their biological functions and evaluates their potential to predict LNM.

The discovery of cuproptosis and ferroptosis mechanisms has introduced new therapeutic strategies for tumors with treatment resistance (Liu and Chen, 2024). Current research on metal-dependent cell death predominantly focuses on leveraging tumor cells’ susceptibility to these pathways for therapy, while studies on the characteristics and functions of tumor cells resistant to metal-dependent cell death remain limited. Based on the expression of cuproptosis- and ferroptosis-related inhibitory genes, this study is the first to identify a distinct subset of tumor cells in OSCC that exhibit resistance to both cuproptosis and ferroptosis. In the case of ferroptosis, this resistance is driven by the accumulation of reactive oxygen species, leading to lipid peroxidation when the reactive oxygen species levels exceed the redox buffering capacity provided by glutathione and GPX4 (Lei et al., 2022; Stockwell, 2022). Studies have demonstrated that the hypoxic tumor microenvironment effectively promotes ferroptosis resistance in solid tumors through a hypoxia-inducible factor 1α-dependent mechanism (Yang et al., 2023). For cuproptosis, cells relying primarily on glycolysis for energy exhibit notable resistance to this pathway (Tsvetkov et al., 2022). Additionally, glutathione has been identified as a protective factor against copper-mediated cytotoxicity (Saporito-Magrina et al., 2018). Our study revealed that during the transition from the Epi_1 to Epi_2 subtype, there is an upregulation of glycolysis, glutathione metabolism, and hypoxia-related pathways, accompanied by reduced reactive oxygen species biosynthesis activity (Figure 2I). These findings further corroborate the copper- and ferroptosis-resistant properties of this unique tumor cell subtype. Moreover, compared to other tumor cell subtypes, Epi_2 is enriched with various tumor progression-related signaling pathways, including epithelial-to-mesenchymal transition pathways associated with metastasis (Figure 2D). This suggests a pivotal role for this specific subtype in the development and progression of OSCC.

Tumor cells with mesenchymal morphology and metastatic states typically exhibit pronounced treatment resistance but tend to be more sensitive to ferroptosis (Zhang H. et al., 2024). In our study, compared to nPT, mPT demonstrated stronger resistance to metal-dependent cell death, with the proportion of Epi_2 cells correlating with LNM. Furthermore, we identified Epi_0, a subtype abundant in mLN, as having the lowest MCDR score (Figure 2F). Epi_0 likely represents a group of metastatic, metal-dependent death-sensitive cells, consistent with existing findings. Additionally, our cell communication analysis revealed that Epi_2 cells exhibited enhanced interactions with endothelial cells, particularly through VEGF and CXCL signaling pathways (Figures 4B–D), suggesting a potential role in promoting endothelial migration and lymphangiogenesis. We hypothesize that Epi_2 in mPT may facilitate LNM. Due to the hypoxic environment in mPT, their lipid metabolism remains unchanged. However, upon reaching distant organs, cancer cells may adopt lipid metabolic characteristics that optimize survival during circulation and extravasation, rendering them more sensitive to metal-dependent cell death (Wang et al., 2024). Notably, our findings highlight a potential link between MCDR and the immune microenvironment. Specifically, our results show a reduced proportion of CD8^+^ Tex cells and a lower abundance of cDC in the Epi2_high group. This suggests that patients with high Epi2 expression may derive less benefit from immunotherapy and may exhibit an immunosuppressive microenvironment. These observations indicate that alternative therapeutic strategies should be considered for this subset of patients. Further investigation is warranted to explore the underlying mechanisms of the reduced cDC content and the formation of the immunosuppressive microenvironment in these patients.

Our study identified GPX4, CDKN2A, and CTSV as characteristic markers of the Epi_2 subtype. GPX4, a key regulator of ferroptosis, has been recognized as a prognostic biomarker for patients undergoing neoadjuvant chemotherapy (Sha et al., 2021), and is associated with the growth and metastasis of gastric adenocarcinoma (Cheng et al., 2024). CDKN2A, encoding the tumor suppressors p16 and p14ARF, is one of the most frequently homozygously deleted genes across all human cancers (Mulvaney, 2023), and acts as an inhibitor of cuproptosis. Studies have shown that CDKN2A expression correlates with lymphocyte infiltration levels in 22 types of pan-cancers and may serve as a biomarker for immune infiltration in cancer (Chen et al., 2021). However, its role in cancer LNM remains underexplored. CTSV is a human lysosomal cysteine protease primarily expressed in the thymus, corneal epithelium, and testes under normal physiological conditions (Sereesongsaeng et al., 2020). It has been found to promote metastasis in lung and renal cell carcinomas (Lin et al., 2020; Zhu et al., 2023). However, its role in OSCC has not been investigated. Our immunohistochemical results showed significantly higher expression of GPX4 and CTSV in mPT compared to nPT (Figures 5A,C). CDKN2A expression was also higher in mPT, though not statistically significant, possibly due to a limited sample size. Fluorescence analysis revealed increased co-expression of these three markers in mPT (Figure 5E), further supporting the potential link between metal-dependent death-resistant cells and LNM.

This study has certain limitations. First, the limited number of tissue samples may explain the lack of significant differences in CDKN2A expression between mPT and nPT. Second, the mechanisms by which Epi_2 promotes LNM were not explored in depth. Furthermore, due to the limited availability of sequencing data in public databases and the challenges in accurately determining LNM status, it is difficult to extract microenvironmental information at different stages of LNM based on data analysis within the framework of the “PUMP” principle. Lastly, the potential reasons behind the resistance to immunotherapy and anti-EGFR targeted therapy in patients with high Epi_2 feature remain uninvestigated.

## 5 Conclusion

This study integrated a large dataset of OSCC single-cell transcriptomes to isolate malignant cells, followed by clustering and functional enrichment analyses. Using ferroptosis- and cuproptosis-inhibitory genes as markers, an epithelial subtype associated with MCDR was identified in mPT. Subsequent spatial transcriptomics, TCGA data analysis, and immunohistochemical experiments confirmed its correlation with LNM. Furthermore, this subtype was linked to resistance to both immunotherapy and anti-EGFR targeted therapies. Doramapimod was identified as a potential therapeutic candidate targeting this subtype. In conclusion, this study provides a novel target for the diagnosis and treatment of OSCC with LNM, offering potential applications for early prediction of LNM and the preservation of functional lymph nodes.

## Data Availability

The original contributions presented in the study are included in the article/Supplementary Material, further inquiries can be directed to the corresponding authors.

## Supplementary material

SUPPLEMENTARY FIGURE S1 InferCNV analysis of malignant epithelial cells in oral squamous cell carcinoma (OSCC). T cells and B cells were selected as reference cells.

SUPPLEMENTARY FIGURE S2 GO functional enrichment analysis of five malignant epithelial cells subtypes. GO enrichment pathways of Epi_0, Epi_1, Epi_2, Epi_3 and Epi_4, including biological process (BP), cellular component (CC) and molecular function (MF).

SUPPLEMENTARY FIGURE S3 KEGG functional enrichment analysis of five malignant epithelial cells subtypes.

SUPPLEMENTARY FIGURE S4 Transcription factor analysis of five malignant epithelial cell subtypes. The first six highly enriched transcription factors of each subtype are shown in the figure.

SUPPLEMENTARY FIGURE S5 Screening of Epi_2 characteristic markers. (A) The expression of top8 differentially expressed genes in Epi_2 in metastatic primary tumor (mPT), non-metastatic primary tumor (nPT) and metastatic lymph node (mLN). (B) The correlation between CTSV and metal-dependent cell death resistance (MCDR) score in malignant cells (P < 0.0001, r = 0.23).

## Abbreviations

AUC, area under curve; CDKN2A, cyclin dependent kinase inhibitor 2A; CTSV, cathepsin-V; cDC, conventional dendritic cells; DEGs, differentially expressed genes; GEO, Gene Expression Omnibus; GO, Gene Ontology; GPX4, glutathione peroxidase four; KEGG, Kyoto Encyclopedia of Genes and Genomes; LN, lymph node; LNM, lymph node metastasis; NK, natural killer; nLN, non-metastatic lymph node; nPT, non-metastatic primary tumor; MDCR, metal-dependent cell death resistance; mLN, metastatic lymph node; mPT, metastatic primary tumor; OM, oral mucosa; OSCC, oral squamous cell carcinoma; PT, primary tumor; RNA-seq, RNA sequencing; scRNA, single-cell RNA; scRNA-seq, single-cell RNA sequencing; TCGA, The Cancer Genome Atlas; Tcm, central memory T cells; Tem, effector memory T cells; Tex, exhausted T cells; Treg, regulatory T cells
