# Supplementary figures and images for: Metal-dependent cell death resistance contribute to lymph node metastasis of oral squamous cell carcinoma
Source: Front Cell Dev Biol. 2025 Feb 27;13:1541582. doi: 10.3389/fcell.2025.1541582 (PMC11903458; doi:10.3389/fcell.2025.1541582)

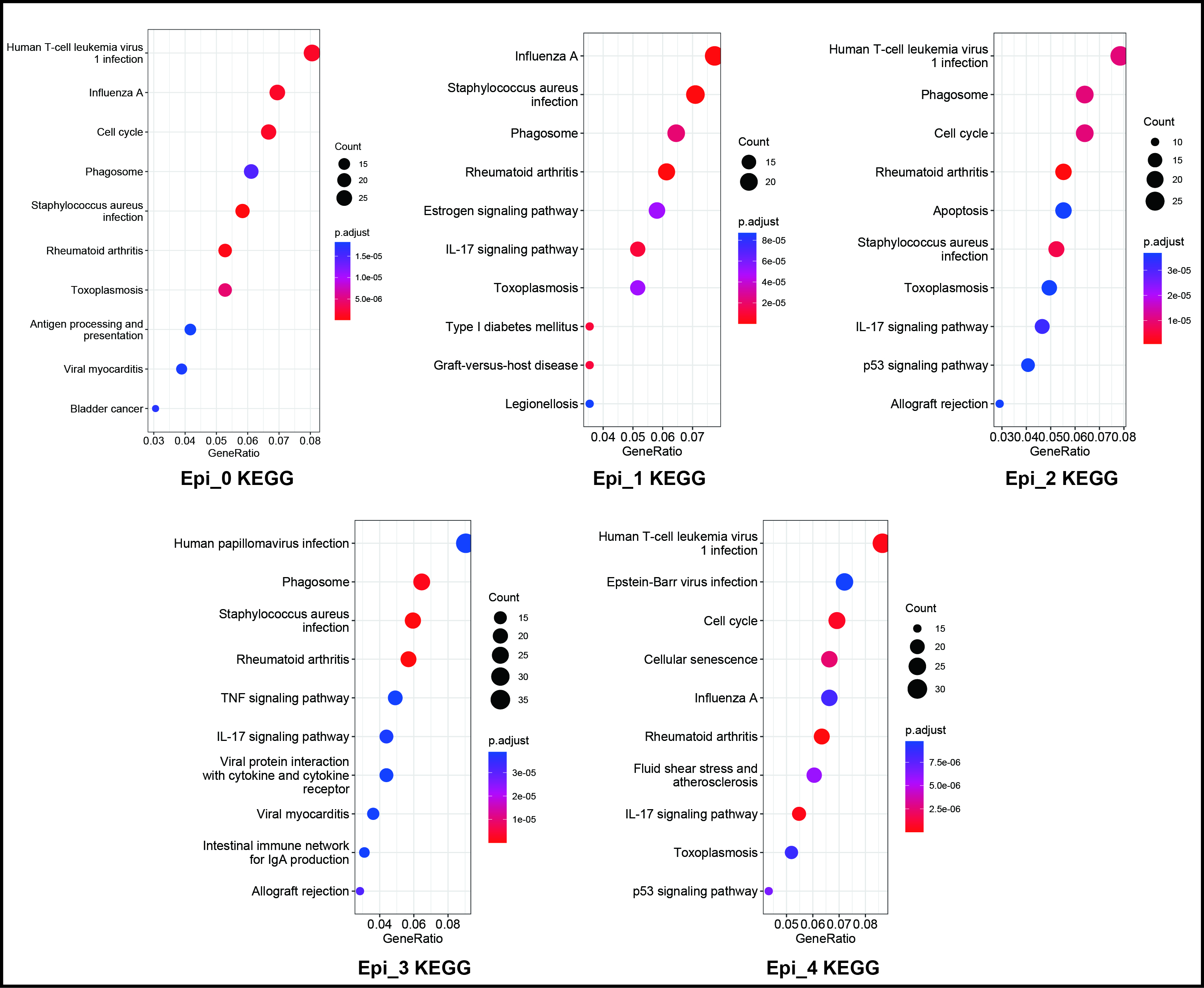

Supplement: Supplementary file 1 [file Image3.jpeg]

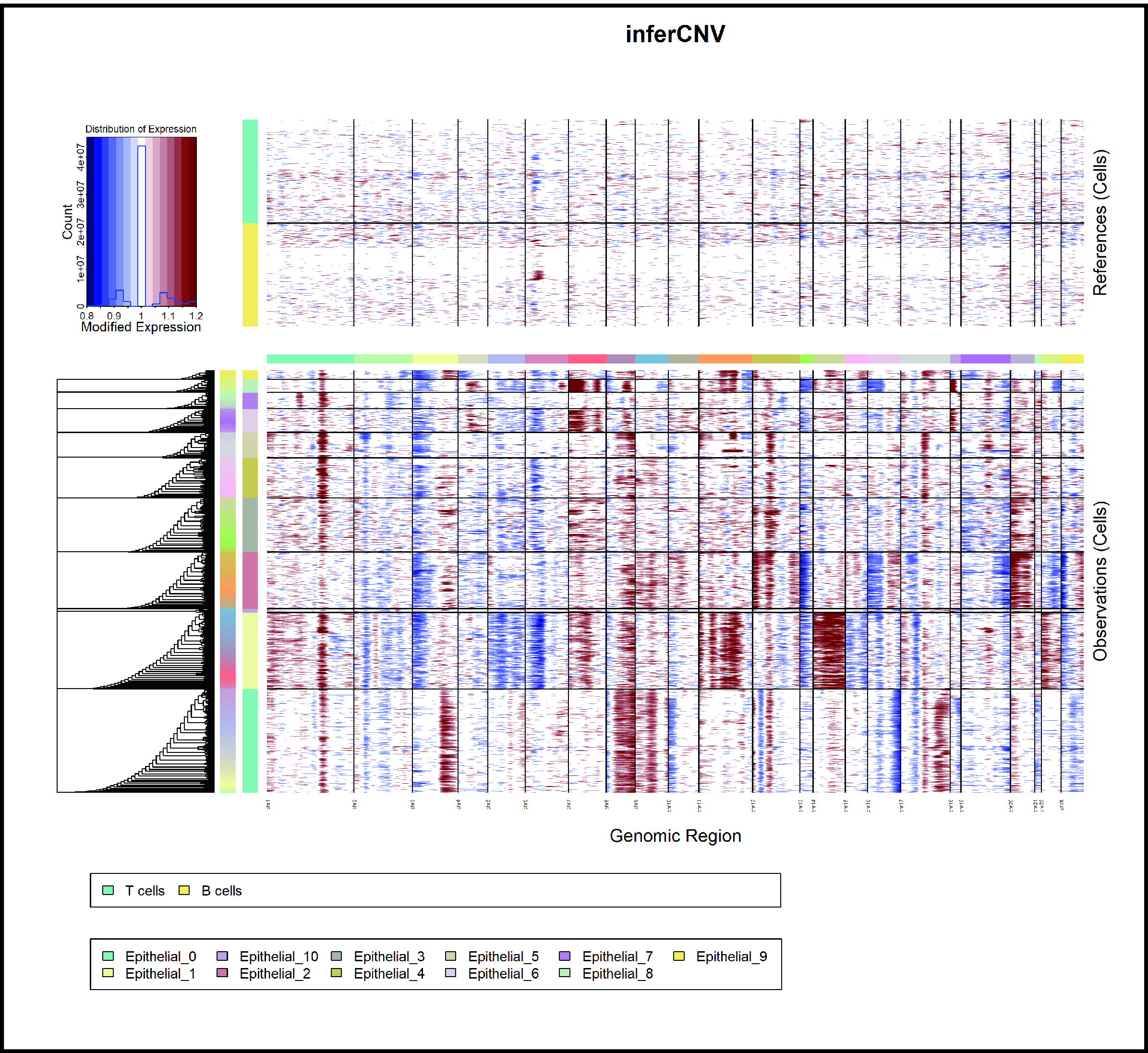

Supplement: Supplementary file 2 [file Image1.jpeg]

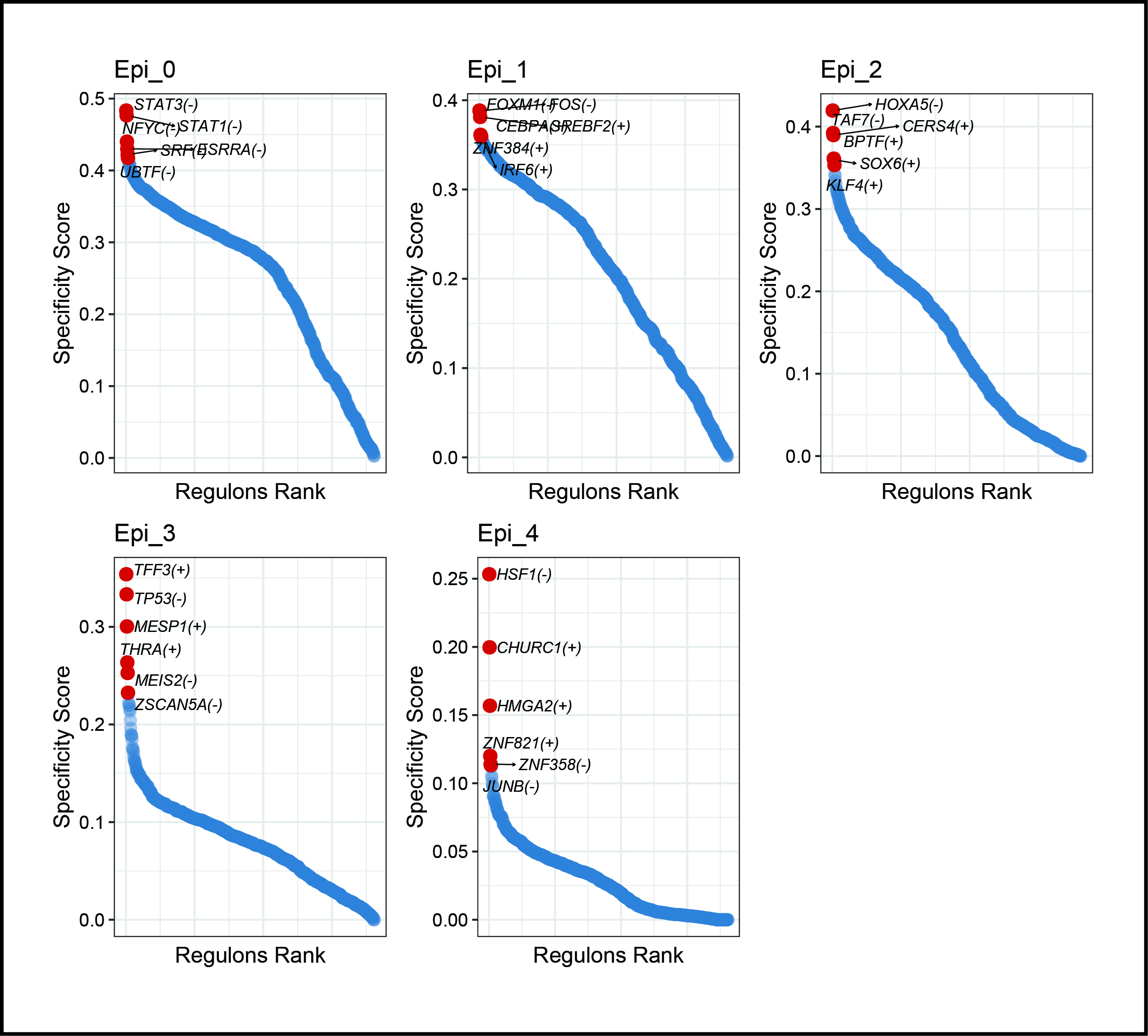

Supplement: Supplementary file 3 [file Image4.jpeg]

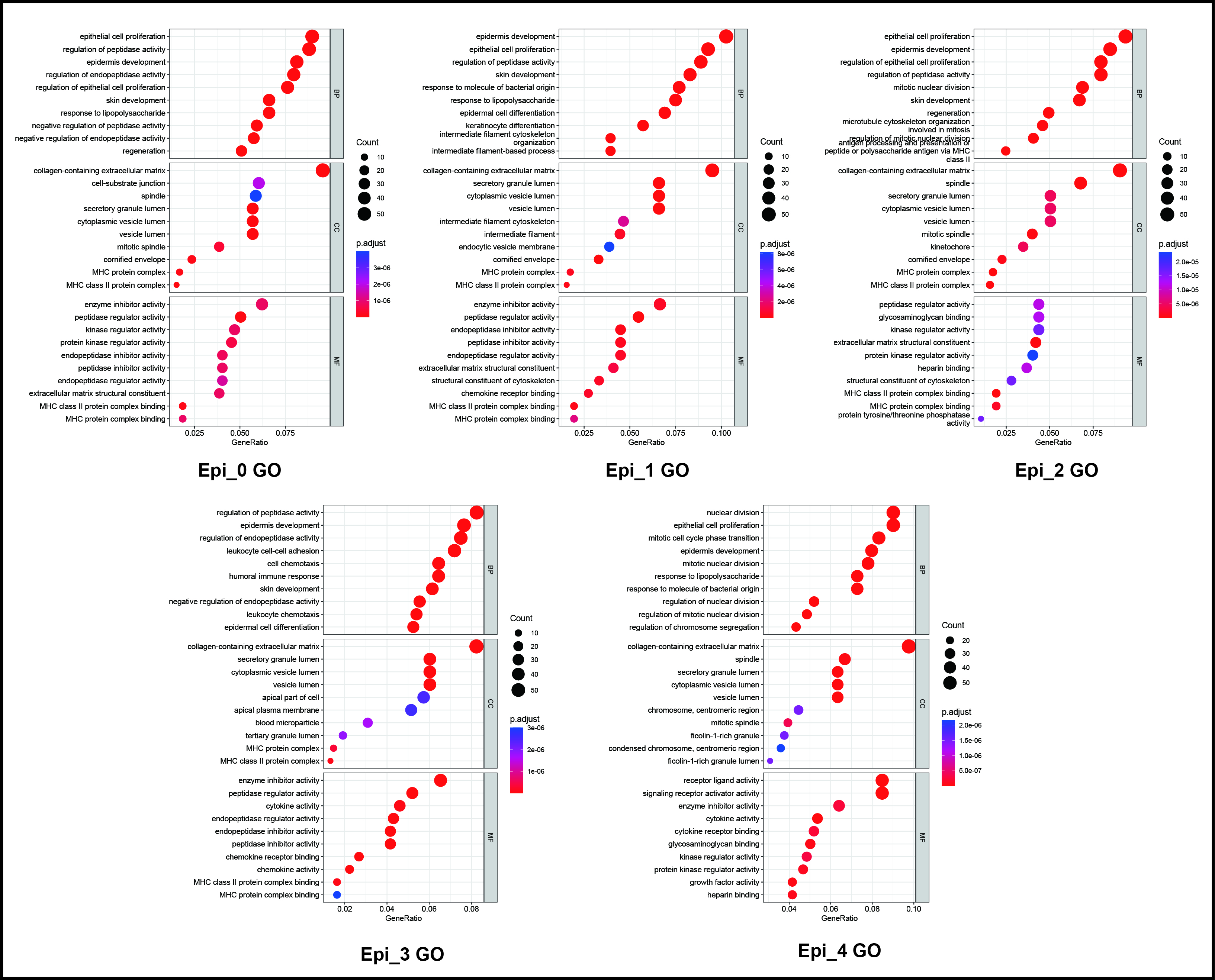

Supplement: Supplementary file 4 [file Image2.jpeg]

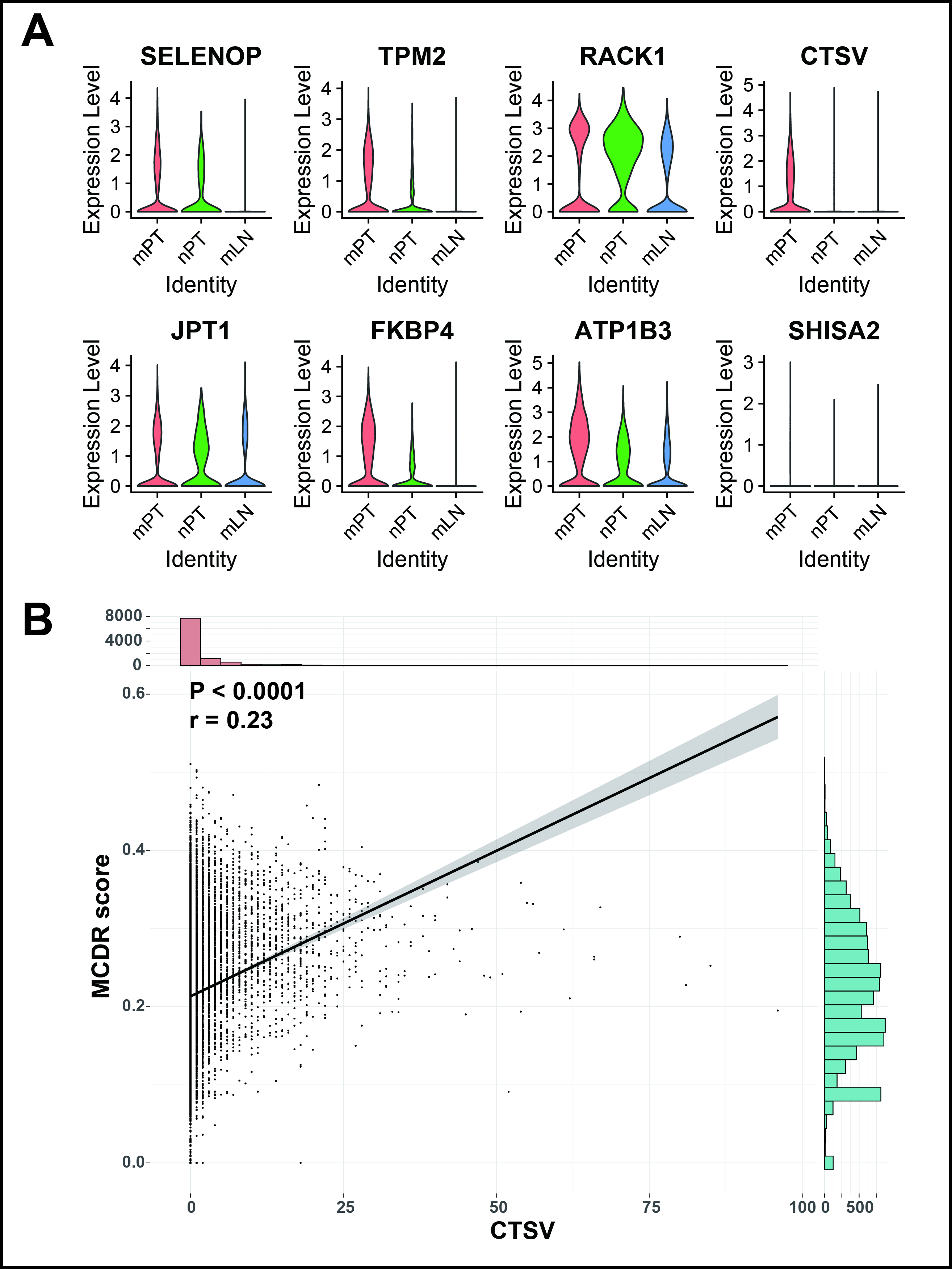

Supplement: Supplementary file 5 [file Image5.jpeg]
